# Bilirubin Molecular Species Play an Important Role in the Pathophysiology of Acute-on-Chronic Liver Failure

**DOI:** 10.3390/ijms25158181

**Published:** 2024-07-26

**Authors:** Stephany M. Castillo-Castañeda, Jacqueline Cordova-Gallardo, Liliana Rivera-Espinosa, Juan L. Chavez-Pacheco, Mariana M. Ramírez-Mejía, Nahum Méndez-Sánchez

**Affiliations:** 1 Liver Research Unit, Medica Sur Clinic & Foundation, Mexico City 14050, Mexico; smcc-fanny@hotmail.com (S.M.C.-C.); mich.rm27@gmail.com (M.M.R.-M.); 2 Medical, Dental and Health Sciences Master and Doctorate Program, National Autonomous University of Mexico, Mexico City 04510, Mexico; 3 Faculty of Medicine, National Autonomous University of Mexico, Mexico City 04510, Mexico; jacquiemex2@yahoo.com.mx; 4 Hepatology, General Surgery Department, General Hospital Dr. Manuel Gea González, Mexico City 14080, Mexico; 5 Pharmacology Department, National Institute of Pediatrics, Mexico City 04530, Mexico; lili_rives@yahoo.com (L.R.-E.); jchavez_pacheco@hotmail.com (J.L.C.-P.); 6 Plan of Combined Studies in Medicine (PECEM-MD/PhD), Faculty of Medicine, National Autonomous University of Mexico, Mexico City 04510, Mexico

**Keywords:** acute-on-chronic liver failure, liver cirrhosis, bilirubin, unconjugated bilirubin, bilirubin monoglucuronide, bilirubin diglucuronide, liquid chromatography–mass spectrometry

## Abstract

Bilirubin plays a key role in early diagnosis, prognosis, and prevention of liver diseases. Unconjugated bilirubin (UCB) requires conversion to a water-soluble form through liver glucuronidation, producing monoglucuronide (BMG) or diglucuronide bilirubin (BDG) for bile excretion. This study aimed to assess the roles of bilirubin’s molecular species—UCB, BMG, and BDG—in diagnosing and understanding the pathogenesis of liver cirrhosis in patients with acute-on-chronic liver failure (ACLF), compensated liver cirrhosis (LC) patients, and healthy individuals. The study included patients with ACLF and compensated LC of diverse etiologies, along with healthy controls. We collected laboratory and clinical data to determine the severity and assess mortality. We extracted bilirubin from serum samples to measure UCB, BMG, and BDG using liquid chromatography–mass spectrometry (LC-MS). The quantification of bilirubin was performed by monitoring the mass charge (*m*/*z*) ratio. Of the 74 patients assessed, 45 had ACLF, 11 had LC, and 18 were healthy individuals. Among ACLF patients, the levels of molecular species of bilirubin were UCB 19.69 μmol/L, BMG 47.71 μmol/L, and BDG 2.120 μmol/L. For compensated cirrhosis patients, the levels were UCB 11.29 μmol/L, BMG 1.49 μmol/L, and BDG 0.055 μmol/L, and in healthy individuals, the levels were UCB 6.42 μmol/L, BMG 0.52 μmol/L, and BDG 0.028 μmol/L. The study revealed marked elevations in the bilirubin species in individuals with ACLF compared to those with compensated cirrhosis and healthy controls, underscoring the progression of liver dysfunction. The correlation of BMG and BDG levels with commonly used inflammatory markers suggests a relationship between bilirubin metabolism and systemic inflammation in ACLF.

## 1. Introduction

Acute-on-chronic liver failure (ACLF) is a syndrome characterized by the acute decompensation of chronic liver disease associated with organ failures and high short-term mortality. ACLF develops shortly after a precipitating event, which is usually related to systemic inflammation caused by infections and acute liver injury. The pathophysiology of ACLF has been closely associated with intense systemic inflammation sustained by circulating pathogen-associated molecular patterns (PAMPs) and damage-associated molecular patterns (DAMPs) [1,2,3]. The development of organ failures may be a result of a combination of tissue hypoperfusion, direct immune-mediated damage, and mitochondrial dysfunction [4]. ACLF is graded into three stages (ACLF grades 1–3) based on the number of organ failures, using an organ assessment failure score that employs some variables like bilirubin [1,2]. As there is an increasing clinical need to assess the prognosis and response to intervention, a key predictor of short-term mortality is needed. In a previous study, we showed that high total and unconjugated bilirubin (UCB) concentrations are independent variables associated with the risk of 1-week mortality [5].

It has been suggested that bilirubin is an important biomarker for early diagnosis, prognosis, disease prevention, and drug response, among others, because of its beneficial effects such as antioxidant, anti-inflammatory, and cytoprotective effects, which occur at physiological or moderately elevated serum concentrations of UCB, 20–170 µM in adults [6,7,8]. The antioxidant role can be explained by its multiple double bonds and a reactive hydrogen atom at C-10, which compete for peroxyl radicals, as demonstrated by Stocker et al. [9]. Another mechanism is based on bilirubin being oxidized to the non-toxic metabolic precursor biliverdin and then recycled back to bilirubin by biliverdin reductase [10,11]. As a cytoprotective role, bilirubin can inhibit pro-apoptotic genes (TNF-, Fas, iNOS, caspase-3, -8, -9, and p38MAPK) and upregulate anti-apoptotic genes (HO-1 and bcl-2) [7]. The cardiovascular benefits of bilirubin derive from lowering serum cholesterol levels, altering the oxidative state of lipid–protein interactions, controlling blood pressure, and directly affecting platelet activation [12]. The anti-inflammatory properties of bilirubin are associated with a variety of mechanisms, such as inhibition of adhesion molecule expression, suppression of inflammatory cell infiltration, reduction of proinflammatory cytokines (TNF-α, IL-6, visfatin, and C-reactive protein [CRP]), and suppression of T-cell proliferation and activation [13]. Bilirubin also exerts immunomodulation by inhibiting the complement cascade (at the binding of the C1 complex to the antibody), altering the phagocytic and antigen-presenting function of macrophages, and suppressing the Th1 effector response by preventing the translocation of NF-κB to the nucleus. In addition, intracellular accumulation of UCB has been shown to reduce IL-2 production by lymphocytes [6]. Nevertheless, despite its beneficial role, bilirubin must be glucuronidated (conjugation of bilirubin with glucuronic acid) in the liver to be excreted in the bile. The conjugation occurs when the enzyme UGT1A1, located mainly in microsomes (soft endoplasmic reticulum, rough endoplasmic reticulum, Golgi membranes, and the nuclear envelope), modifies one or both -COOH groups of bilirubin by covalently bonding with glucuronic acid to form monoglucuronide (BMG) or diglucuronide bilirubin (BDG) [14,15,16].

The aim of this study was to evaluate the performance of the molecular species of bilirubin: UCB, BMG, and BDG, and their role in the diagnosis and pathogenesis of liver cirrhosis patients with ACLF compared to patients with compensated liver cirrhosis and healthy individuals.

## 2. Results

### 2.1. General Description of the Groups

A total of 74 consecutive patients were studied: 45 were diagnosed with ACLF and 11 with compensated liver cirrhosis, and there were 18 healthy individuals. ACLF manifested in patients with a median age of 52 years (IQR 47–58), while healthy individuals had a median age of 46 years (IQR 26–59), and those with compensated cirrhosis had a median age of 63 years (IQR 53–71). Of the ACLF patients, 73% were male and 27% were female. For compensated cirrhosis patients, 36% were male and 64% were female. Finally, among the healthy individuals, 67% were male and 33% were female. Most patients with ACLF were enrolled during the first day of hospitalization, including those admitted to the emergency department or intensive care unit. The main etiology of cirrhosis was alcohol-associated liver disease (ALD): 64.4% in patients with ACLF and 45.5% in the compensated cirrhosis group. Table 1 shows the results of all the variables measured and the calculated scores for each group.

### 2.2. Clinical Characteristics ACLF Patients

According to the EASL-CLIF Consortium definition, which includes precipitating events, complications, and organ failure, 40% of patients were hospitalized for bacterial infection, 33% had gastrointestinal bleeding, 11% had drug-induced liver injury, 5% had active alcoholism, 3.4% had another precipitating event, and 10.3% had no precipitating event. The most common medical complication was sepsis (29%), followed by esophageal varices (27%), hepatic encephalopathy (13%), ascites (13%), peritonitis, acute renal failure, and alcoholic hepatitis (7%, 4%, and 7%, respectively). By calculating the CLIF-C ACLF score, the type and severity of organ failure were determined, as well as the specific ACLF grade for each patient. Of the 45 patients with ACLF, 43 developed at least one or more organ failures. Renal failure was the most common organ failure in 20 of 43 patients (46.5%), followed by liver failure in 19 patients (44.1%), cerebral in 13 patients (30.2%), coagulation failure in 10 (23.2%), and circulatory and respiratory failures in 8 patients each (18.6%) (Table 2). Patients with ACLF were graded; 2 patients (5%) were classified as NO ACLF, 18 (40%) as ACLF 1, 16 (35%) as ACLF 2, and 9 (20%) as ACLF 3. Table 3 shows the features of the patients according to each grade.

### 2.3. Identification and Quantification of Bilirubin Molecular Species

**Figure 1:**
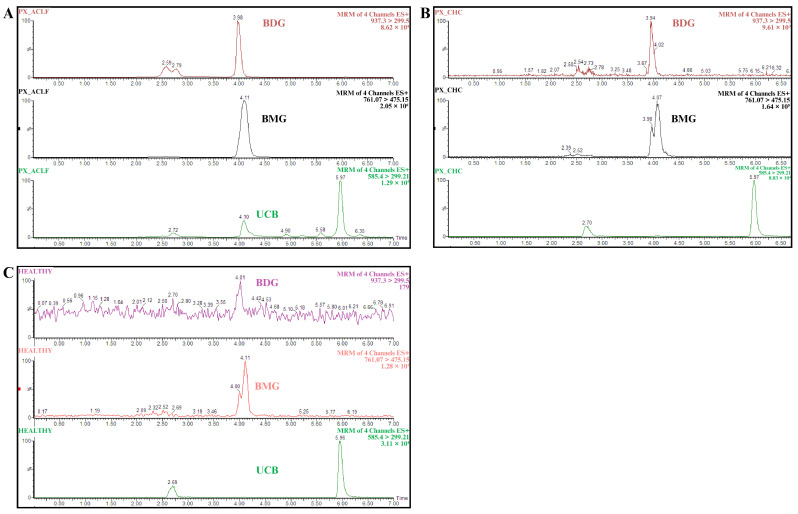
Spectrograms for each group analyzed. Panel (**A**–**C**) show the molecular species obtained by multiple reaction monitoring chromatograms of individual channels. Samples were collected and processed from (**A**) patients with acute-on-chronic liver failure, (**B**) patients with hepatic encephalopathy, and (**C**) healthy volunteers. The ionic transitions *m*/*z*^1+^ were 585.4 > 299.2 for UCB, 761.3 > 475.3 for BMG, and 937.3 > 299.5 for BDG. UCB, unconjugated bilirubin; BMG, bilirubin monoglucuronide; BDG, bilirubin diglucuronide.

The molecular species of the bilirubin were identified by mass-to-charge ratio (*m*/*z*), and their retention times were as follows: the peak at 5.97 min was assigned to UCB, at 4.11 min to BMG, and at 3.98 min to BDG. The LC-MS spectrogram of one patient from each group is shown in Figure 1, showing the fragmentation spectra for UCB, BDG, and BMG. The peak signals of each can be observed.

In the healthy control group, bilirubin molecular species concentrations were measured as follows: 6.42 μmol/L for UCB, 0.52 μmol/L for BMG, and 0.028 μmol/L for BDG. Within the group of patients with compensated cirrhosis, the mean levels recorded were 11.29 μmol/L for UCB, 1.49 μmol/L for BMG, and 0.055 μmol/L for BDG. For patients in the ACLF group, the values were significantly higher: 19.69 μmol/L for UCB, 47.71 μmol/L for BMG, and 2.120 μmol/L for BDG. The differences between the groups were statistically significant with *p*-values of 0.000 (Table 4). The ratios of BMG/UCB and BDG/UCB were calculated for each group, and significant differences were observed between them (*p*-value = 0.000 for both ratios; Supplementary Table S1). The ACLF patients exhibited higher BMG/UCB and BDG/UCB ratios compared to the compensated LC patients and healthy individuals. This finding indicates that patients with ACLF have significantly elevated concentrations of these biomarkers, which reflect the greater extent of liver damage and dysfunction in this group.

For each ACLF grade, the median levels of UCB, BMG, and BDG were 36.08, 9.86, and 0.621 μmol/L for NO ACLF patients; 20.42, 32.50, and 1.434 μmol/L for ACLF 1; 23.67, 75.88, and 1.285 μmol/L for ACLF 2; and 18.31, 144.23, and 5.311 μmol/L for ACLF 3. The differences between the grades were statistically significant for BMG and BDG with a *p* value of 0.007 and 0.009, respectively, while not significant for UCB (Supplementary Table S2).

### 2.4. Bilirubin Molecular Species in Relation to Inflammation

UCB, BMG, and BDG were correlated with leukocytes and C-reactive protein, the most commonly used biomarkers of inflammation. It was found that there was a moderately positive correlation between BMG and BDG with C-reactive protein (CRP) (r = 0.523 and r = 0.527) and leukocytes (r = 0.446 and r = 0.407) with significant *p*-values (0.000, 0.000, 0.000, and 0.000), meaning that as leukocytes increase so do BMG and BDG, as well as CRP. However, UCB was not correlated with any of the inflammatory biomarkers (Supplementary Table S3).

### 2.5. Bilirubin Molecular Species Performance as Factors Associated With Mortality and ACLF Development

**Figure 2:**
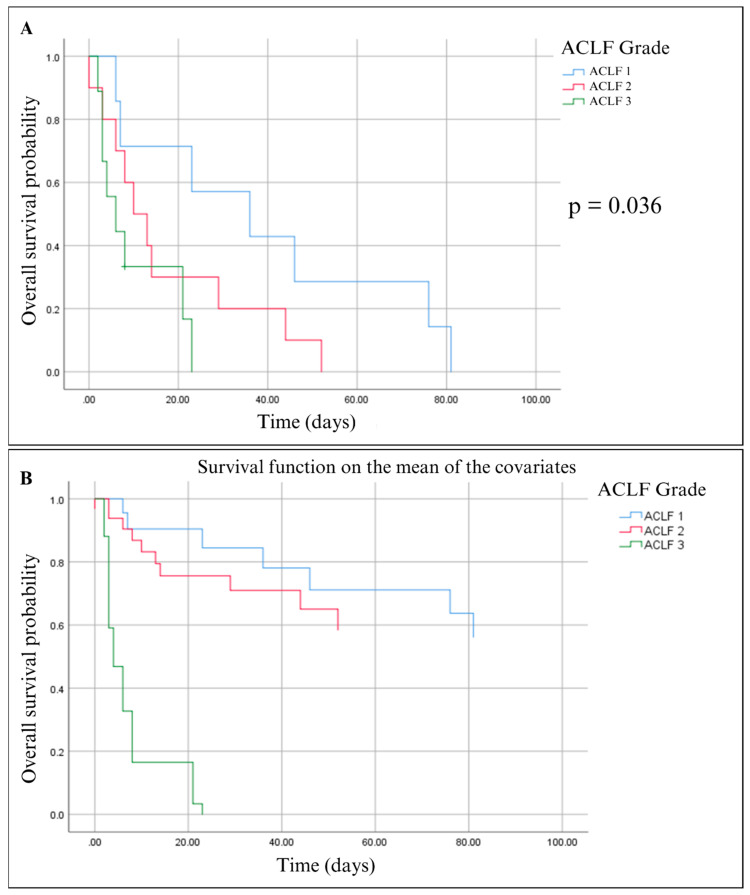
Kaplan–Meier Survival Analysis of ACLF Grades. (**A**) Kaplan–Meier plot for the three ACLF grades compared by log rank test *p* < 0.036. (**B**) Kaplan–Meier plot for the three ACLF grades analyzed by cox regression including the variables BMG, UCB, MELD, Child–Pugh, and total bilirubin.

In our study, Kaplan–Meier analysis estimated a median survival of 39 days for ACLF 1, 18 days for ACLF 2, and 10 days for ACLF 3, while the overall survival was 22 days. This can be interpreted as the time it takes 50% of the population to reach the event, but for the total population who died, the graph showed that the mortality of patients classified by ACLF grade had differences, ACLF 1 died at 80 days, ACLF 2 at 55 days, and ACLF 3 at 20 days; the log rank test showed a *p*-value of 0.016, indicating a statistically significant difference between groups (Figure 2A).

In the Cox regression analysis, we found that BMG was statistically significant (0.017) as a prognostic factor for mortality as total bilirubin, MELD, and Child–Pugh score (0.004, 0.027, and 0.001) (Table 5). Cumulative survival was plotted according to ACLF grade using the mean of the covariates and showed that patients with ACLF 1 and ACLF 2 had a survival rate of 60% while ACLF 3 had a survival rate of 0% (Figure 2B).

**Figure 3:**
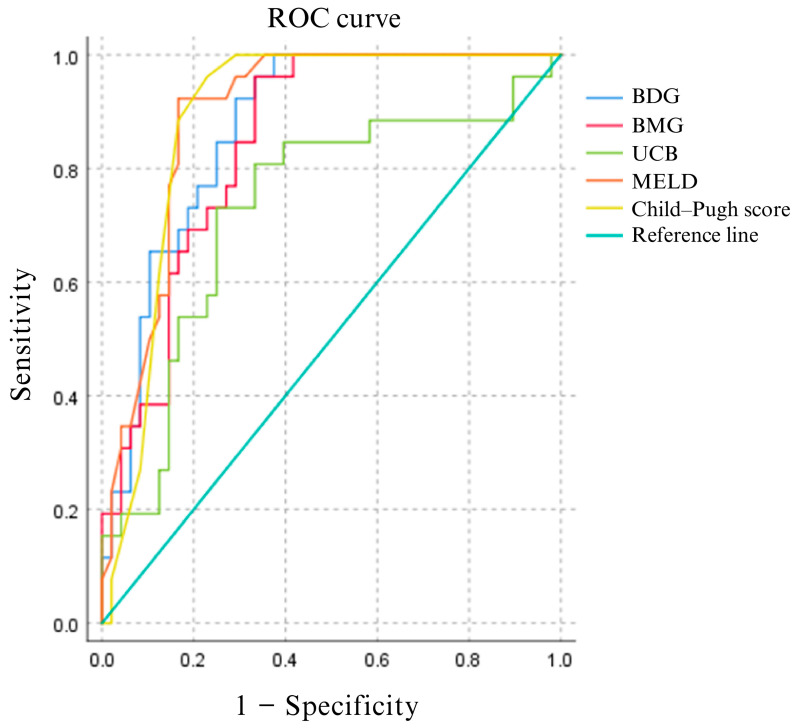
Receiver Operating Characteristic (ROC) curves for overall survival. The graph shows the performance of three molecular species of bilirubin, namely BDG, BMG, and UCB, alongside the MELD and Child–Pugh scores. This visual representation helps to understand the value of sensitivity and specificity in assessing liver function and predicting outcomes.

The molecular species of bilirubin were analyzed using ROC analysis with MELD and Child–Pugh scores (Figure 3). Cut-off values were selected based on the best sensitivity and specificity. Therefore, we can assess the prognosis of a patient with a serum concentration of 17.50 μmol/L for UCB, 13.13 μmol/L for BMG, and 0.770 μmol/L for BDG (Supplementary Table S4).

## 3. Discussion

Significant variations in bilirubin levels were observed among patients with compensated cirrhosis and those with ACLF; patients in the ACLF group presented markedly elevated levels of BMG, followed by UCB. It was expected that there would be a significant discrepancy between the results observed in patients with liver disease and those observed in healthy subjects. In order to mitigate the impact of significant deviations due to the substantial differences in concentrations, ratios were used to normalize these results. However, the results remained statistically significant. The ratios allow for the identification of specific biochemical patterns associated with disease progression, as evidenced by the elevation of molecular species of bilirubin, which becomes even more pronounced when assessing bilirubin levels in the different grades of ACLF. This suggests a correlation between bilirubin species, BMG and BDG, and disease severity, revealing possible pathophysiological mechanisms such as impaired bilirubin metabolism, conjugation, and excretion. Since BMG and BDG are products of bilirubin conjugation processes, their elevated levels in the bloodstream could indicate a decreased ability of the liver to conjugate and excrete bilirubin. Normally, the ratio of monoglucuronide to diglucuronide in serum is 1:1, but this ratio was not observed in our study. This decreased formation of BDG and increased levels of BMG may be associated with decreased hepatic UGT activity. This deterioration is underscored by elevated UCB levels, signaling impaired liver function. Furthermore, the positive correlation between BMG, BDG, and inflammatory markers such as CRP and leukocytes further solidifies the role of these bilirubin species as indicators of systemic inflammation in liver disease. Inflammation is a critical component of the pathophysiology of ACLF; in our study, it was observed that bacterial infection was the major precipitating event of ACLF, and it is well known that bacteria can induce inflammation via PAMPs and virulence factors. PAMPs are recognized by the host via specific receptors called pattern recognition receptors (PRRs). The binding of PRRs leads to the stimulation of signaling cascades. These cascades activate transcription factors that can induce a number of genes encoding molecules involved in inflammation [1].

Furthermore, molecular species of bilirubin may be a useful marker in patients with liver failure. This study is the first to quantify BMG and BDG (conjugated bilirubin) as well as unconjugated bilirubin (UCB), providing with greater accuracy the ratio of both. These are typically quantified through the diazo reaction, forming the compound azobilirubin, which is a pigment and can be measured spectrophotometrically at 450 nm. However, they are not directly quantified in the manner employed in our work. Consequently, the concentrations of these analytes are estimated from stoichiometry or mathematical calculations that infer the concentration. ROC analysis was used to demonstrate this utility by comparing it with the most commonly used scores, and the AUC was used to evaluate it. If the AUC is greater than 0.5, it is a strong diagnostic technique, but it must be greater than 0.8 to be considered acceptable [17]. BDG and BMG were found to have almost the same AUC as Child–Pugh and MELD scores, indicating good diagnostic accuracy, with slightly lower performance of UCB. From the prognostic perspective, BMG was found to be the molecular species of bilirubin most closely related to patient survival, along with other prognostic scores. This was evidenced by the Kaplan–Meier survival analysis, which showed a marked contrast in survival rates between patients with liver failure, who had a median survival of 40 days, and those without, who had a median survival of 80 days. As liver dysfunction worsens, bilirubin concentrations increase due to the decreased ability of the liver to metabolize bilirubin, suggesting an important role for bilirubin in the underlying mechanisms of liver failure.

As more bilirubin is released into the system, more toxicity occurs, causing membrane and mitochondrial dysfunction, oxidative stress, apoptosis, inflammation, and even epigenetic modifications in susceptible organs. All these bilirubin-induced toxic mechanisms can exacerbate multiorgan dysfunction as evidenced by the number of organ failures. The organ failure was determined by the CLIF Consortium Organ Failure (CLIF-C OF) score [18], the most common organ affected was the kidney. Renal excretion is the main route of elimination for the less water-soluble UCB, this is consistent with reports that bilirubin accumulates in renal tubular cells, increases membrane permeability, impairs mitochondrial function leading to oxidative stress, and disrupts renal tubular architecture by developing acute tubular necrosis and apoptosis. This leads to excessive sodium chloride delivery to the macula densa, promoting glomerular vasoconstriction and retention of H_2_O and Na^+^, minimizing perfusion and causing ascites [8,19]. The second organ affected was the liver, as reflected by the higher levels of BMG found in ACLF patients. Cerebral failure was also a predominant organ failure, as it is well known that UCB can be a neurotoxin due to its lipid solubility, and it can cross the blood–brain barrier and affect the membranes and mitochondria of neurons, microglial cells, and astrocytes, resulting in their activation, impaired myelination, and neuronal death. The main consequence of bilirubin neurotoxicity is the occurrence of encephalopathy [20,21].

This study presents several strengths. First, it has a robust sample comprising a diverse group of participants: individuals with ACLF, those with compensated liver cirrhosis, and healthy individuals. This diversity allows for a detailed comparative analysis across liver disease stages and health states, providing insight into disease mechanisms and how variations in liver function affect clinical outcomes. Second, the use of advanced analytical techniques such as LC-MS for in-depth characterization of bilirubin molecular species significantly improves biochemical analysis. Finally, a comprehensive follow-up of all participants until hospital discharge or death ensures a complete data set, essential for a robust and comprehensive analysis of the results and of the impact of molecular bilirubin species.

Nevertheless, we are aware of the issues that were not addressed and the limitations of the study. First, as this was the first study on ACLF and molecular bilirubin species, the sample size was not calculated, which may lead to bias. In addition, during the course of our study, we invited numerous volunteers to participate in the compensated LC and healthy individual groups. Some volunteers accepted the invitation, but subsequent analysis of their samples for biochemical testing revealed that they did not meet the required criteria, resulting in their exclusion and a reduction in the number of analyzable samples. In addition, an unforeseen failure of the chromatograph resulted in the loss of additional samples and reduced the number of available samples for analysis. Second, although internal validation was performed, future studies should perform external validation of the results obtained. Third, we were not able to measure proinflammatory cytokines or to perform molecular studies that would help us to delve deeper into the effect of molecular bilirubin species on the pathophysiology of ACLF. Finally, further studies should evaluate UCB, BMG, and BDG in the classification of other liver diseases, such as decompensated cirrhosis, hepatic encephalopathy, and acute hepatitis, with different observation periods before the model can be put into wider clinical use.

## 4. Material and Methods

This study is registered on ClinicalTrials.gov under record number NCT05566548.

### 4.1. Patients’ Enrollment

Patients diagnosed with liver cirrhosis who developed ACLF were recruited from General Hospital ‘Dr. Manuel Gea González’ and Hospital Medica Sur; written informed consent was obtained from each patient included in the study. Additionally, patients with compensated liver cirrhosis and a control group of healthy individuals were included. Prior to enrollment, written informed consent was obtained from either the patients themselves or their legal representatives. The study was open to both male and female participants aged between 20 and 70 years. Recruitment was carried out consecutively and gradually increased until a statistically significant sample size was achieved. The study protocol conforms to the ethical guidelines of the 1975 Declaration of Helsinki, as reflected in the a priori approval of the institution’s human subjects research committee (protocol code 2021-EXT-637 approved by the Ethics Committee of Medica Sur Clinic & Foundation and protocol code 04-54-2022 approved by the Ethics Committee of General Hospital ‘Dr. Manuel Gea González’).

### 4.2. Diagnostic Criteria and Associated Mortality for Each Group

The ACLF patients were diagnosed according to the definition of the European Association for the Study of the Liver-Chronic Liver Failure (EASL-CLIF) consortium [22] on admission to the emergency room or intensive care unit. Their clinical record was reviewed to assess the etiology of cirrhosis, previous episodes of ACLF, and/or complications. Patients with liver cirrhosis due to any cause, such as alcoholic liver disease (ALD), chronic hepatitis C virus (HCV), metabolic-associated fatty liver disease (MAFLD), and autoimmune liver conditions, were enrolled in the study. Patients were excluded if they had concomitant human immunodeficiency virus (HIV) infection, biliary liver disease, history or presence of extrahepatic malignancies, liver transplant, or severe systemic or psychiatric disease.

Upon admission, blood samples were collected from patients with ACLF for biochemical testing. These tests include total bilirubin, direct bilirubin, indirect bilirubin, total protein, albumin, ALA, AST, GGT, ALP, LDH, glucose, creatinine, Hb, RBC, WBC, platelets, PT, INR, and aPTT. The tests were conducted on the same day as the sample collection on the DxC 700 AU clinical chemistry system (Beckman Coulter, Brea, CA, USA). Prognosis scores (MELD, MELD-Na, Child–Pugh, ALBI) were calculated and mortality was recorded for all patients in the study, as all patients were followed until hospital discharge or death. The group with cirrhosis was recruited at their medical consultation to ensure that they had compensated cirrhosis, during which a blood sample was taken to perform the previously mentioned biochemical tests. The sample of healthy individuals was obtained at the Research Tower of the National Institute of Pediatrics, after signing the informed consent of the subjects participating in the study. The healthy individuals were evaluated using a clinical questionnaire to assess health status, including BMI, blood pressure, medical history, and normal physical examination.

### 4.3. Analytical Methods

#### Analysis of Bilirubin Molecular Species in Serum by LC-MS

After patient enrollment, a blood sample was collected and centrifugated to obtain serum, all samples were put in black envelopes to avoid light. Sample processing was performed through liquid–liquid extraction with methanol and DMSO (80:20 *v*/*v*), and the separation and identification of bilirubin molecular species was performed by ultra-performance liquid chromatography (UPLC) using a Quattro micro-API detector (Waters, Milford, MA, USA) in an electropositive ionization mode (ES+) with multiple reaction monitoring (MRM) for the analytes to be detected. We followed the methodology of Putluru et al. [23] with some modifications, using a binary gradient mobile phase of 5 mM ammonium acetate, pH 6.8 (mobile phase A), and ACN-acetonitrile (mobile phase B) at a flow rate of 0.300 mL/min. The chromatographic separation of the analytes was performed on an Acquity BEH C18 column (2.1 × 100 mm, 1.7 µm). For analyte identification, the *m*/*z* of each molecular species was monitored, *m*/*z* 585 to 299 for bilirubin, *m*/*z* 761 to 475 for BMG, and *m*/*z* 937 to 122 for BDG. MassLynx software (version 4.1) was used for data acquisition and analysis. The analytical method employed in the study was validated in accordance with NOM-177-SSA1-2013 [24], which is harmonized with international guidelines for the validation of analytical methods, including those set forth by the FDA and the EMA [25,26]. Using the concentration results, we created a ratio of BMG and BDG divided by UCB for all the patients.

### 4.4. Statistical Analysis

Univariate analysis was performed to compare the LC and HS groups with the ACLF group using the Mann–Whitney U test, and all groups were compared using the Kruskal–Wallis test for data with non-normal distributions, while the χ2 test or Fisher’s exact test was used for qualitative variables. All *p* values < 0.05 were considered significant. Bilirubin molecular species were correlated with the most common variables reflecting inflammation (WBC and Protein C Reactive). For ACLF grade, Kaplan–Meier plots were used to construct survival curves, and the log-rank test was used to analyze differences between grades. Cox regression was used to determine whether the molecular species of bilirubin were independent prognostic factors and the most commonly used prognostic scores. The method was adjusted using a stepwise backward method based on the inclusion or exclusion of variables. Risks were estimated using 95% confidence intervals (95% IC) and their corresponding *p* values were calculated. Receiver-operating characteristic (ROC) curves were used to determine the diagnostic accuracy and mortality prognosis of bilirubin molecular species by determining the cut-off point at which the highest sensitivity and specificity are achieved and comparing them with the MELD score, Child Pugh score, and total bilirubin by analyzing the area under the ROC curve (AUC). Data analysis was performed using SPSS (version 27.0; SPSS, Chicago, IL, USA).

## 5. Conclusions

In conclusion, this study has provided evidence that molecular bilirubin species, UCB, BMG, and BDG, serve as significant biomarkers in the assessment of ACLF. The study revealed marked elevations in these bilirubin species in individuals with ACLF compared to those with compensated cirrhosis and healthy controls, underscoring the progression of liver dysfunction. This impairment is further evidenced by the correlation of BMG and BDG levels with inflammatory markers, suggesting a deeply intertwined relationship between altered bilirubin metabolism and systemic inflammation in the context of ACLF. These data point to underlying pathological processes that exacerbate liver disease, where bilirubin not only serves as a byproduct of altered liver function, but also potentially contributes to the inflammatory milieu characteristic of ACLF. The prognostic value of these bilirubin species was demonstrated, with BMG emerging as a particularly strong predictor of mortality, along with established clinical scores such as MELD and Child–Pugh. These findings reinforce the utility of bilirubin molecular species as indicators of liver disease severity and suggest the potential for their use in guiding clinical decision-making. Future studies are encouraged to validate these biomarkers and explore their role in the pathophysiology of liver diseases, which could lead to better prognostic tools and therapeutic strategies.

## Figures and Tables

**Table 1:** Sociodemographic, clinical, and laboratory characteristics at baseline.

Characteristics	Healthy Individuals (*n* = 18)	Liver Cirrhosis Compensated (*n* = 11)	ACLF (*n* = 45)	*p* Value
**Demographics**				
Sex	Men: 12 (67%) Women: 6 (23%)	Men: 4 (36%) Women: 7 (64%)	Men: 33 (73%) Women: 12 (27%)	
Age	46 (26–59)	63 (53–71)	52 (47–58)	
Patient diagnosed on			Hospitalization: 14 (31%) Emergency: 31 (69%)	
**Etiology**				
Alcohol-associated liver disease (ALD)	-	45.5%	64.4%	
Autoimmune hepatitis	-	9.1%	8.9%	
Hepatitis C virus	-	18.2%	8.9%	
DILI	-	9.1%	2.2%	
MAFLD	-	18.2%	15.6%	
**Biochemical parameters**				
Total bilirubin (mg/dL)	0.5 (0.5–0.8)	1.05 (0.52–1.21)	7.76 (3.725–20.165)	0.000
Direct bilirubin (mg/dL)	0.1 (0.1–0.2)	0.32 (0.12–0.41)	4.37 (1.53–15.75)	0.000
Indirect bilirubin (mg/dL)	0.4 (0.3–0.6)	0.67 (0.38–0.83)	3.43 (2.07–6.91)	0.000
Total Proteins (g/dL)	7.3 (6.775–7.625)	7.3 (7.01–7.5)	5.96 (5.2–6.40)	0.000
Albumin (g/dL)	4.3 (3.875–4.5)	3.94 (3.02–4.1)	2.52 (2.22–2.95)	0.000
ALT (IU/L)	37.5 (27.8–43.5)	28 (17–52)	39 (21–70)	0.558
AST (IU/L)	33 (28.5–41.75)	53 (29–100)	76 (44–147)	0.000
GGT (IU/L)	39 (23.75–52.5)	115 (26–193)	124 (94–247.5)	0.000
ALKP (IU/L)	96.5 (85–114)	142 (118–280)	120 (84–205.5)	0.010
LDH (IU/L)	151 (143.25–199.75)	167 (147–195)	216 (171.5–304)	0.002
Glucose (mg/dL)	93 (88.75–103)	100 (84–115)	105 (92–140.5)	0.060
Creatinine (mg/dL)	0.75 (0.6–0.9)	0.81 (0.7–1.01)	1.75 (0.97–2.79)	0.000
Erythrocytes (10^6^/µL)	4.75 (4.475–5.3)	3.7 (3.37–4.26)	2.75 (2.47–3.80)	0.000
Hemoglobin (g/dL)	15.25 (13.82–16.27)	10.8 (9.46–13.9)	9.7 (7.85–12.45)	0.000
Platelets (10^3^/µL)	225 (200.75–292.5)	128 (92–152)	96 (62–141)	0.000
Leucocytes (10^3^/µL)	6.53 (5.11–7.3175)	4.7 (3.8–5.8)	9.3 (6.15–14.8)	0.000
Prothrombin time (PT) (s)	12.1 (11.4–12.5)	13.4 (11.9–14.9)	20.15 (15.47–27.87)	0.000
INR	1.05 (0.99–1.09)	1.19 (1.06–1.33)	1.92 (1.39–2.46)	0.000
Activated partial thromboplastin time (aPTT) (s)	28.1 (26.5–30.1)	31.3 (29.9–32.7)	36.9 (32.8–42.8)	0.000
C-reactive Protein (mg/dL)	1.65 (1.09–2.00)	2.46 (1.87–3.01)	5.85 (4.01–14.70)	0.000
Na (mEq/L)	139 (138–140)	138 (137–141)	131 (126–137)	0.001
K (mEq/L)	4.2 (4.1–4.6)	4.1 (3.8–4.6)	4.3 (3.65–4.75)	0.945
Cl (mEq/L)	105 (103.5–107)	107 (104–108)	100 (95–105.75)	0.032
Ca (mg/dL)	9.5 (9–9.65)	8.29 (7.57–8.79)	7.97 (7.5–8.5)	0.006
Phos (mg/dL)	3.8 (3.65–4.2)	3.39 (2.87–3.58)	3.75 (2.98–5.03)	0.376
Mg (mg/dL)	1.90 (1.80–1.95)	1.85 (1.80–1.97)	2.1 (1.80–2.70)	0.236
MELD score	7 (7–7.25)	9 (8–11.25)	26.5 (20.25–33.75)	0.000
MELD-Na score	8 (7–9)	-	26.5 (21–35.75)	0.002
Child–Pugh score	4 (4–5)	5 (5–5.25)	10 (9–11)	0.000
ALBI score	−3.01 (−3.08–−2.6775)	−2.395 (−2.8675–−1.56)	−0.92 (−1.245–−0.345)	0.000

**Table 2:** Clinical characteristics and organ failures of ACLF patients.

**Precipitating event**	
Bacterial infection	18 (40%)
Gastrointestinal bleeding	15 (33%)
Drug-induced liver injury (DILI)	5 (11%)
Active alcoholism within the past 3 months	2 (5%)
No precipitating event	4 (9%)
Another precipitating event	1 (2%)
**Medical complications**	
Sepsis	13 (29%)
Peritonitis	3 (7%)
Hepatic encephalopathy	3 (13%)
Ascites	6 (13%)
Esophageal varices	12 (27%)
Acute renal injury	2 (4%)
Alcoholic hepatitis	3 (7%)
**Number and types of organ failures**	
Number total of organ failure	43/45 (95.6%)
One organ failure	18/43 (42%)
Two organ failures	16/43 (37%)
Three organ failures or more	9/43 (21%)
**Type of liver failure**	
Liver failure	19/43 (44.1%)
Renal failure	20/43 (46.5%)
Cerebral failure	13/43 (30.2%)
Coagulation failure	10/43 (23.2%)
Circulatory failure	8/43 (18.6%)
Respiratory failure	8/43 (18.6%)

**Table 3:** Biochemical characteristics of patients with ACLF classified by grade.

Characteristics	No ACLF (*n* = 2)	ACLF 1 (*n* = 19)	ACLF 2 (*n* = 18)	ACLF 3 (*n* = 6)	*p* Value
**Sex**	H: 57%	H: 80%	H: 90%	H: 33%	
**Age**	53 (39–56)	54 (50–60)	52 (45–59)	53 (43–63)	
**Biochemical parameters**					
Total bilirubin (mg/dL)	1.1 (0.49–1.71)	5.69 (2.77–17.56)	6.53 (4.41–12.81)	22.15 (16.26–29.78)	0.004
Direct bilirubin (mg/dL)	0.27 (0.19–0.36)	2.46 (1.39–9.72)	3.77 (1.52–6.82)	17.72 (7.73–23.88)	0.003
Indirect bilirubin (mg/dL)	0.82 (0.3–1.35)	3.23 (1.23–7.21)	3.3 (2.36–5.06)	4.44 (3.4–7.98)	0.051
Total Proteins (g/dL)	5.42 (4.68–6.17)	5.98 (5.33–6.49)	6.08 (5.3–6.91)	5.12 (4.51–5.85)	0.116
Albumin (g/dL)	2.65 (1.79–3.51)	2.48 (2.05–3.36)	2.535 (2.31–3)	2.41 (2.24–2.57)	0.736
ALT (IU/L)	25.5 (21–30)	35 (19–75.5)	24.5 (20.3–62.8)	73 (60–93.5)	0.027
AST (IU/L)	29.5 (26–33)	75 (55.5–110.5)	54.5 (38–96)	192 (140–236)	0.001
GGT (IU/L)	128 (102–155)	122 (55–370)	116 (71–174)	147 (101–266)	0.588
ALKP (IU/L)	84 (80–88)	118 (86–219)	135 (79–184)	120 (92–227)	0.622
LDH (IU/L)	159.5 (109–210)	213 (159.5–250.5)	227.5 (173.5–307.25)	211 (163–362)	0.530
Glucose (mg/dL)	168 (135–201)	118 (89–173)	102 (87.75–137.75)	102 (91.5–120.5)	0.237
Creatinine (mg/dL)	0.93 (0.6–1.27)	1.44 (0.78–2.48)	1.97 (1.63–2.77)	2.78 (1.46–6.12)	0.086
Erythrocytes (10^6^/µL)	3.61 (3.11–4.11)	3.01 (2.64–3.99)	2.63 (2.38–3.59)	2.64 (2.32–3.745)	0.319
Hemoglobin (g/dL)	10.97 (9.54–12.4)	10.1 (8.2–13.15)	8.8 (7.7–10.85)	10.2 (7.8–12.4)	0.715
Platelets (10^3^/µL)	117 (63–171)	112 (73–150.5)	69.5 (46.5–114)	108 (76–153)	0.225
Leucocytes (10^3^/µL)	8.05 (7.2–8.9)	7.6 (5.3–15.4)	9.15 (5.62–13.92)	14.1 (7.4–18.55)	0.606
Prothrombin time (PT) (s)	12.4 (11.3–13.5)	18.6 (14.05–27.6)	20.4 (16.12–28.2)	24.7 (18.15–35)	0.082
Activated partial thromboplastin time (aPTT) (s)	1.11 (1.01–1.21)	2 (1.25–2.44)	1.83 (1.43–2.57)	2.26 (1.63–3.165)	0.125
INR	27.05 (25.2–28.9)	36.9 (35.6–41.1)	37.35 (30.9–47.375)	39.6 (34.05–48.75)	0.216
C-reactive Protein (CRP)	-	5.245 (2.10–9.51)	6.45 (4.41–16.09)	6.9 (4.90–9.07)	0.315
Na	-	135.5 (130.75–137.25)	130.5 (125.75–140)	124.5 (121.75–131)	0.014
K	-	4.4 (3.6–4.65)	4.4 (3.77–5.1)	4 (3.3–4.7)	0.388
Cl	-	102 (96–106.25)	100 (93.75–107.25)	92.5 (85.25–101.25)	0.184
Ca	-	8.05 (7.56–8.52)	8.20 (7.65–9.2125)	7.78 (7.51–8.17)	0.132
Phos	-	3.56 (2.72–4.05)	4.17 (2.84–6.69)	4.1 (2.95–5.98)	0.563
Mg	-	2 (1.77–2.57)	2.3 (1.87–2.62)	2.4 (1.6–3.05)	0.661

**Table 4:** Molecular species of bilirubin concentration for each patient group.

	Healthy Individuals (18)	Liver Cirrhosis Compensated (11)	ACLF (45)	*p* Value
UCB (μmol/L)	6.42 (4.90–8.75)	11.29 (8.28–17.69)	19.69 (14.14–41.42)	0.000
BMG (μmol/L)	0.52 (0.34–0.76)	1.49 (0.43–5.41)	47.71 (14.54–105.56)	0.000
BDG (μmol/L)	0.028 (0.020–0.033)	0.055 (0.036–0.076)	2.120 (0.873–6.260)	0.000

**Table 5:** Results of Cox regression of different prognostic scores.

Variable	RR	95% CI	*p* Value
Multivariate analysis			
MELD	1.080	1.009–1.155	0.027
Child–Pugh	1.688	1.249–2.283	0.001
BMG	1.009	1.002–1.017	0.017
UCB	0.996	0.979–1.012	0.595
Total bilirubin	0.892	0.825–0.964	0.004

## Data Availability

The original contributions presented in the study are included in the article/supplementary materials; further inquiries can be directed to the corresponding author/s.

## Supplementary Materials

The following supporting information can be downloaded at: https://www.mdpi.com/article/10.3390/ijms25158181/s1.

## Abbreviations

aPTT, Activated partial thromboplastin time; ALT, Alanine aminotransferase; ALBI, albumin-bilirubin score; ALD, alcohol-associated liver disease; ALP, Alkaline phosphatase; AST, Aspartate aminotransferase; BMG, Bilirubin monoglucuronide; BDG, Bilirubin diglucuronide; BMG, Body Mass Index; RBC, Erythrocytes; GGT, Gamma glutamyl transpeptidase; Hb, Hemoglobin; INR, International normalized ratio; LDH, Lactate dehydrogenase; WBC, Leukocytes; LC, liver cirrhosis; MRM, multiple reaction monitoring; PT, Prothrombin time; UCB, Unconjugated bilirubin; UGT1A1, Uridine diphosphate-glucuronosyltransferase 1A1.
