# Supplementary material for: Bilirubin Molecular Species Play an Important Role in the Pathophysiology of Acute-on-Chronic Liver Failure
Source: Int J Mol Sci. 2024 Jul 26;25(15):8181. doi: 10.3390/ijms25158181 (PMC11311833; doi:10.3390/ijms25158181)
Supplement: Supplementary file 1 [file ijms-25-08181-s001.zip › ijms-3043797-supplementary.pdf]

Supplementary Tables

Supplementary Table S1: Ratios calculated of the BMG and BDG

Ratio BMG/UCB				Ratio BDG/UCB			
**Healthy Individuals**	**Compensated Liver Cirrhosis**	**ACLF**	**Valor p**	**Healthy Individuals**	**Compensated Liver Cirrhosis**	**ACLF**	**Valor p**
0.0039 (0.0022–0.0090)	0.0045 (0.0031–0.0429)	0.1204 (0.0345–0.2513)	0.000	0.0747 (0.0559–0.1027)	0.1316 (0.0738–0.3192)	1.8030 (0.9151–6.2681)	0.000

Supplementary Table S2: Molecular species of bilirubin concentration for each ACLF grade

	**No ACLF**	**ACLF 1**	**ACLF 2**	**ACLF 3**	**p value**
UCB (µmol/L)	36.08 (9.91–62.254)	20.42 (12.02–58.09)	23.67 (14.03–42.02)	18.31 (16.98–26.98)	0.964
BMG (µmol/L)	9.86 (6.67–13.06)	32.50 (12.46–64.46)	75.88 (14.81–165.63)	144.23 (54.42–158.26)	0.019
BDG (µmol/L)	0.621 (0.375–0.867)	1.434 (0.855–6.178)	1.285 (0.725–7.472)	5.311 (2.385–7.221)	0.073

Supplementary Table S3: Correlation between biomarkers of inflammation and the molecular species of bilirubin

	**C-Reactive Protein**		**Leukocytes**	
	r	***Valor p***	***r***	***Valor p***
UCB	0.208	0.114	0.139	0.239
BMG	0.523	0.000	0.446	0.000
BDG	0.527	0.000	0.407	0.000

Supplementary Table S4: Results of the ROC curve analysis for the variables analyzed to predict mortality in ACLF patients.

Variables	**AUC**	**95 % CI**	***p*-value**	**Cut-off values**		
				**Sensitivity**	**1 – Specificity**	**Concentration (µmol/L) or score**
UCB	0.731	0.604–0.857	0.000	0.731	0.292	17.50
BMG	0.846	0.761–0.931	0.000	0.846	0.292	13.13
BDG	0.872	0.794–0.950	0.000	0.846	0.292	0.770
Child Pugh score	0.888	0.810–0.965	0.000	0.885	0.167	10
MELD score	0.894	0.823–0.966	0.000	0.923	0.167	22
